# Psychological construction as a theoretical principle for guiding cognitive-behavioral treatments

**DOI:** 10.3389/fpsyg.2024.1363819

**Published:** 2024-03-19

**Authors:** Alexandru I. Tiba

**Affiliations:** Department of Psychology, University of Oradea, Oradea, Romania

**Keywords:** psychological construction, cognitive behavior therapy (CBT), essentialism, emotional disorders, constructed mind

Cognitive behavior therapies (CBT) are considered the benchmark for evidence-based psychological treatments for psychological disorders (David et al., 2018). CBT treatments work and are well-known because they constantly update with new scientific research encompassing the theory, models, and their real-life applications (Ingram et al., 2019). The integration of psychopathology research into the development of CBT models and practices is the current standard for enhancing their scientific plausibility (Hayes and Hofmann, 2018). Although CBT is characterized by a strong integration of science, it considers emotion and cognition as essential entities that exist as natural kinds and that we can identify, assess, and change in order to improve people's lives. In other words, it is based on an essentialist assumption regarding conscious psychological states. Recent research suggests a different perspective, claiming that psychological categories are not “essential” phenomena, but observer-dependent constructed entities (Barrett, 2009; Gündem et al., 2022).

In this article, I argue that (1) psychological essentialism is one core assumption in CBT theories and models; (2) psychological essentialism has been recently challenged by a “psychological construction approach” of psychological states (Barrett, 2009); and (3) a “psychological construction approach” brings significant changes to the practice of CBT.

## Psychological essentialism and the “essence” of psychological states in CBT

Essentialism suggests that categories we encounter, such as dogs, trees, or birds, have an underlying essence or existence that causally determines what they are (Brick et al., 2022; Neufeld, 2022). Psychological essentialism is the hypothesis that psychological categories (emotions, cognitions, and behaviors) are natural kinds rather than social constructions (Barrett, 2009; Brick et al., 2022). They have an essence that determines their characteristics and what they are (Brick et al., 2022; Neufeld, 2022). They are distinct (emotion is separate from cognition) and homogenous (types of emotion share more attributes between them than with types of cognition) categories with sharp boundaries (Neufeld, 2022). For instance, feeling sad is a conscious psychological state that is caused by having an emotion. Emotions such as sadness exist as natural kinds. When we feel sad, we experience the reality of the emotion of sadness. Feeling sad is an attribute of sadness. Low arousal, body feelings, avoidant action tendencies, and sad thoughts are accompanying features of sadness, determined by its essence, which is emotion. It is distinct from cognition and behavior. It has distinct brain bases and causal relationships. Thus, we try to recognize, discover, and study emotions.

There are two major arguments that suggest CBT is founded on a psychological essentialist assumption. The first major argument for the psychological essentialism of CBT is the fact that CBT considers perception, cognition, emotion, and behavior as distinct entities that are discovered based on assessments of their manifestations. Based on the assessment of their attributes, the therapists identify and discover which cognitions, behaviors, and emotions are involved in patients' problems (Westbrook et al., 2007). Both therapists and patients should not confuse emotion, behavior, and cognition. Emotion, behavior, and cognition have distinct attributes that reflect their essence (Neufeld, 2022). When patients “mistakenly” say, “I feel like a failure,” the therapists have to correct patients to recognize it is a thought, not an emotion, and to help them realize they are confounding feelings with cognitions. Thus, CBT bears heavily on “faculty” psychology and the essentialist assumption that emotion, cognition, and behavior are distinct, homogenous entities with clear boundaries that exists as natural kinds and can be discovered (Neufeld, 2022). The second major argument for the psychological essentialism of CBT is the ABC model of cognitive causation in CBT. The ABC model asserts that emotions (C) are not caused by A (negative events), but by beliefs or cognition (B) (Westbrook et al., 2007). One entity, cognition, causes another entity, emotion, or behavior. Thus, the ABC model relies on the essentialist assumption that these entities are natural kinds that interact based on mechanistic causation (Barrett, 2009). A natural kind (“cognition”) causes another natural kind (“emotion”) (Barrett, 2009). All these descriptions of the CBT principles suggest a strong essentialist foundation for CBT.

## The psychological construction approach

Recently, increasing research from affective neuroscience supports the idea that psychological states we know (emotion, cognition, and behavior) are not natural kinds, but conceptual constructions reflecting how we explain what the internal and external sensations stand for given prior experience (see Gündem et al., 2022 for reviews of the evidence). Perception is the mental states humans have when they understand what the external sensations stand for based on prior experience (Barrett, 2009). Cognition refers to the mental state during the process of replaying past experiences in the brain (Barrett, 2009). Emotion refers to mental states when individuals comprehend the meaning of internal bodily experiences (Barrett, 2009). In short, mental states are *ad-hoc* conceptualizations of internal and/or external sensations based on simulations of what those sensations stand for given prior experience (Barrett, 2009). Although there are many differences brought in by the psychological construction approach regarding mental states (Barrett, 2009), here I describe two of them in relation to psychological treatments: the constructed and “recipe-like” nature of mental states and the probabilistic causation. First, psychological essentialism considers conscious mental states as natural kinds (entities with distinct brain bases, features, and mechanisms that control them) (Barrett, 2009). The psychological construction approach negates this view and considers psychological states to be composite “recipe-like” constructions made up of basic ingredients such as concepts, core affect, behavioral repertoire, prior experience, and internal and external sensations (Barrett, 2009). Therefore, the conceptualization and the type of ingredients recruited for its composition determine how a psychological state will change. When we change depressed feelings, distorted emotional concepts and unhelpful ingredients will be of interest. Second, psychological essentialism advances mechanistic psychological causation (Barrett, 2009). A causes B. If we activate or develop an entity A, then we will change another entity B. Instead, the psychological construction approach advanced a probabilistic, not mechanistic causality relationship (Barrett, 2009). The occurrence of cognition does not directly cause emotion. Rather, the presence of a specific cognition increases the likelihood of a constructed state of cognition transitioning into a specific constructed state of emotion (Barrett, 2009). Believing that failing at an exam is awful (B) does not directly cause a feeling of anxiety but increases the probability that the psychological state we have will transform into a state of anxiety (C) rather than just fear.

Previous proposals focused on the clinical implications of a psychological construction approach based on brain-based mechanisms such as dysfunctions of energy regulation in mental disorders (Shaffer et al., 2022). Here, I outlined several consequences of applying the psychological construction approach as a principle for changing mental states through the talking methods of CBT.

## Implications for CBT formulation

Formulation is the process by which we describe the psychological mechanisms that underlie psychological issues and the ways through which we modify them (David et al., 2018). From the psychological construction approach, understanding the patients' emotional problems will require understanding why the patients construct the psychological state they have into dysfunctional feelings of depression and not into functional feelings of sadness. During this process, the therapists will try to find which are the emotional concepts of the individual and which are the ingredients, or “psychological primitives,” of their depressed feelings (i.e., conceptual granularity, prior experience, core affect, behavioral repertories). Then, they will try to find the prior states that increased the probability of having depressed feelings (i.e., beliefs) and the mechanisms that control the frequent construction of their state as depressed feelings (i.e., context, attention). As the therapists explain the relationship between B and C, they will follow a probabilistic causation approach. For instance, instead of teaching the client that his beliefs that the exam is awful cause his feelings of anxiety, based on a psychological construction approach, the therapist will say, “You understand that as long as you believe that failing the exam is awful, you will probably feel anxiety and not concern.”

## Implications for CBT practice

Although traditional CBT treats cognition, behavior, and emotion as distinct entities, at a closer look at the practice of CBT, we may find several precursors of the psychological construction approach. For instance, in the mainstream practice, therapists who follow a behavioral tradition often conceptualize cognition as a behavior and instead of cognitive interventions, to change cognition, they use behavioral interventions. Consequently, rather than employing cognitive restructuring, the therapists alter cognition by changing the environmental cues that initiate cognition (e.g., refraining from remaining in bed if that is the place where negative thinking occurs more frequently), and substituting it with an alternative cognition (e.g., rather than believing “I am useless,” the patients may be asked to recall instances of successful performance, what to change and that one failure does not define us) that has comparable consequences (e.g., motivating and giving importance to the problems).

Treating cognition as a behavior resembles the psychological construction approach.

However, the mainstream approach views cognition as behavior or belief, influenced by different theories on how cognition is understood (cognitivist vs. behaviorism), rather than being the same psychological state differently constructed by the individual as cognition or behavior. The psychological construction approach suggests that the same psychological state may be cognition, behavior, or emotion, depending on how it is constructed by the individual and on its “ingredients.” Thus, changing a cognition as a behavior is not something that follows the theoretical orientation of the therapist, but something that follows how the individual constructs that psychological state. From the psychological construction approach, when patients say I keep thinking “I am useless,” they are referring to a behavior. Probably, this psychological state is predominantly under the control of what is known as behavioral mechanisms (e.g., reinforcement). The client's statement, “I believe I am useless,” might indicate a different construction that the patients have engaged in, that of belief. Then, the intervention will focus on the analysis of confirmatory and dis-confirmatory information to change beliefs. Nonetheless, the individuals may construct the psychological state as something they feel—“I feel I am useless.” In this case, changing the psychological state as a feeling by validating, expressing, and processing the feeling may be more appropriate. As individuals may construct their mental state as different psychological states, depending on the type of construction (emotion, cognition, behavior), we may find some control mechanisms to be more often involved than others. Although early applications of multiple change strategies for the same mental state indicates possible benefits, scientific research may clarify which strategy or combination of strategies and in which condition would be more efficient for a particular individual. Furthermore, the intervention should consider the principles related to the ingredients or “recipe-like” composition of psychological states. When we target changing a psychological state (distorted appraisals) that may transform into dysfunctional feelings, the affective ingredients become the focus of treatment (Tiba, 2010; Tiba and Manea, 2018). For instance, the cognitive satiation procedure is one good illustration of changing the affective ingredients of negative thoughts and reducing their emotional impact. For this purpose, the therapist may introduce a semantic satiation method: “One way to reduce the affective load of the thought “I am useless” is to use semantic satiation. In this exercise, we must repeat the expression “I am useless” more than 40 times until we load the thought with a phonetic rather than affective composition.”

## Summary and outlook

A psychological construction approach brings important changes to how we deliver evidence-based psychological treatments: (1) understanding emotion, cognitive states, and behavior as different constructions of conscious psychological states; (2) changing psychological states is done by changing the general and specific control mechanisms involved in the specific unfolding of those states; (3) changing the ingredients involved in psychological states is a way of changing the relation between “thoughts” and “feelings.” As these principles may be viewed as super-ordinate principles guiding the models of psychological treatments, they can be integrated into the metacognitive principles of the CBT models. Given the enrichment of CBT interventions, the psychological construction approach has the potential to bring significant advancements to CBT treatment.

## Author contributions

AT: Writing—original draft, Writing—review & editing.
